# Spendception: The Psychological Impact of Digital Payments on Consumer Purchase Behavior and Impulse Buying

**DOI:** 10.3390/bs15030387

**Published:** 2025-03-19

**Authors:** Naeem Faraz, Amna Anjum

**Affiliations:** 1 Sino British College, University of Shanghai for Science and Technology, Shanghai 200093, China; naeem.faraz@sbc.usst.edu.cn; 2 International Cultural Exchange School (ICES), Donghua University, West Yanan Road 1882, Shanghai 200051, China

**Keywords:** Spendception, impulse buying, consumer purchase behavior, digital payments, genders’ moderation, mediation, SEM, EFA, CFA

## Abstract

This study introduces a novel construct, Spendception, which conceptualizes the psychological impact of digital payment systems on consumer behavior, marking a significant contribution to the field of consumer psychology and behavioral economics. Spendception reflects the reduced psychological resistance to spending when using digital payment methods, as compared to cash, due to the diminished visibility of transactions and the perceived ease of payments. This research aims to explore the role of Spendception in increasing consumer purchase behavior, whereas the role of impulse buying has been observed as a mediator. To test the proposed model, an extensive survey was performed by collecting 1162 respondents from all walks of life to get the real picture. We employed exploratory factor analysis (EFA) and confirmatory factor analysis (CFA) to validate the measurement of key constructs. To test the hypothetical relations among all the variables, we employed structural equation modeling (SEM). Furthermore, a machine learning technique was used to test the robustness of the model. Results showed that Spendception greatly boosted the consumer purchase behavior, with impulse buying partially mediating the relation. Gender was found to moderate the relationship, with female consumers being more susceptible to impulse buying caused by Spendception. The study showed that digital payment systems made buying feel less noticeable, which led to people spending more without realizing the financial impact. This study introduces Spendception, a novel construct that extends existing consumer behavior theories by explaining how digital payment systems reduce psychological barriers to spending. It bridges the gap between Spendception and the pain of paying, demonstrating that the lack of immediate visibility and physicality in digital payments alters consumers’ perceptions of spending, leading to impulse buying and higher purchase behavior. The findings also offer actionable insights for marketers in designing targeted campaigns that leverage the psychological effects of Spendception. The findings provide actionable insights for marketers to design targeted campaigns and for policymakers to promote financial literacy, ensuring ethical use of digital payment systems.

## 1. Introduction

Over the course of several decades, digital payments have brought about a revolution in global business (Cai et al., 2024; Yang, 2024; S. Zhao et al., 2024a, 2024b). This change began with the introduction of credit cards in the 1950s and 1960s, which let customers make transactions without the need for cash, which played a vital role in economic success (Xu et al., 2024). With the advent of electronic fund transfers (EFTs), point-of-sale (POS) systems, and automated teller machines (ATMs) in the 1970s, transactions became more streamlined and secure (Mariana et al., 2025). The 1990s witnessed the proliferation of the internet, which resulted in the emergence of e-commerce and online payment systems, which were pioneered by software applications such as PayPal. Mobile payment systems and contactless technologies, such as near-field communication (NFC), came into existence in the 2000s.

China’s digital payments journey is unique due to its rapid adoption and general use. The decade of the 1990s saw the establishment of UnionPay, an interbank network that was supported by the government and used to create the framework for digital transactions (Guo et al., 2025; Liu et al., 2024; Wani et al., 2025). The beginning of the twenty-first century saw the proliferation of e-commerce platforms, such as Alibaba, which introduced Alipay in 2004 as a safe and reliable online payment solution (T. Li et al., 2024; M. Zhao & Abeysekera, 2024). The year 2013 marked the beginning of the revolution in mobile payments, which was initiated by WeChat Pay. This application incorporated payment capabilities into Tencent’s messaging service, utilizing QR codes to facilitate transactions in a simple manner. With Alipay and WeChat Pay controlling more than 90 percent of the market, China transformed into a society that was almost entirely cashless by the late 2010s (Alrawad et al., 2023; X. Li et al., 2023; Mombeuil & Uhde, 2021; Pang & Ruan, 2023; Zarco et al., 2024). Beyond the realm of payments, these platforms have expanded their offerings to encompass services, such as investments, loans, and payments for utility bills.

China has leapfrogged traditional payment systems by migrating directly to mobile payments powered by QR codes (Borzova, 2024). This is in contrast to the gradual growth of digital payments that globally followed, beginning with credit cards and progressing to mobile and real-time payments. In China, the government has taken a more aggressive role, as evidenced by the development of the digital yuan, making its system unique in terms of its scale and creativity, as it is environmentally friendly (Xu et al., 2024).

The adoption of digital payment systems has reshaped consumer spending behavior by significantly altering how individuals perceive and interact with money. In traditional cash transactions, the physical exchange of money serves as a tangible reminder of spending, evoking what is often termed the “pain of paying”. This physical and psychological visibility acts as a natural barrier to overspending. However, digital payment methods, such as mobile wallets, credit cards, and online banking, lack this tactile element, creating a psychological detachment from expenditures. This detachment not only reduces the perceived visibility of spending but also fosters behavioral and emotional changes that lead to increased purchases.

To encapsulate the psychological and behavioral shifts brought about by the invisibility and ease of digital payments, this study introduces Spendception as a novel construct. It is a blend of “spend” and “perception”. The term Spendception effectively combines the act of spending with the psychological perception of it. It is a unique terminology that encapsulates the behavioral and psychological aspects of spending through digital payments. The term is catchy, aligns with existing psychological constructs, and adds a modern, research-focused touch. Spendception serves as the central independent variable in this research, capturing the interplay of four critical dimensions: psychological visibility of spending, perceived spending control, ease of digital payment, and emotional detachment.

## 2. Concept of Spendception

Spendception is conceptualized as a multidimensional framework that explains how digital payment systems influence consumer behavior. It reflects the diminished psychological resistance to spending when using digital payment methods compared to cash.

### 2.1. Psychological Visibility of Spending

When we talk about psychological visibility of spending, we are talking about how we think and feel about our financial transactions. When you pay with cash, the act of handing over the money provides a physical reminder of how much you spent (Schomburgk et al., 2024). As cash in a wallet decreases, it serves as a clear and instant sign of spending, making people more aware and reinforcing the mental “cost” of purchases. This level of public awareness serves as a deterrent, making people think more carefully about their financial choices and supporting smart spending. Digital purchases, on the other hand, make this much harder to see. There is a feeling of being disconnected from real financial outflows when using credit cards, mobile apps, or online platforms to make purchases. This is because digital transactions are not tangible.

### 2.2. Perceived Spending Control

Perceived spending control is the feeling that you have control over your money that comes from digital payments being so easy to use. When you pay with cash, you physically hand over the money and see your resources go down. With digital payments, you do not get that instant and tactile feedback. This separation weakens the mental link between spending and its results, which leads to a false sense of control (Davydenko & Peetz, 2024). The speed and ease of digital transactions add to this impression, as the smooth process reduces the mental load that comes with making financial decisions.

### 2.3. Ease of Digital Payment and Emotional Detachment

Detachment is about the changes that happen in our minds and feelings because of how easy and intangible digital transactions are (Ly & Ly, 2024). Digital payments are quick and easy, and you do not even have to do anything to make them happen. You can pay with a card tap, an application, or an automatic withdrawal from your digital bank. This smooth process gets rid of a lot of the problems that come with traditional cash payments, like having to count bills, get change, or hand over cash. This means that buying is less conscious and more automatic, which means that less thought is needed during a transaction. The fact that there is no physical exchange with digital purchases makes them even less personal. When you pay with cash, the act of handing over the money provides a physical link to the transaction that makes the emotional “cost” of spending stronger. Digital transactions, on the other hand, make the trade less clear, which makes it harder for people to connect the transaction with losing money. This generalization lessens the “pain of paying”, which is a psychological effect that happens when people have to part with money (Ly & Ly, 2024).

## 3. Theoretical Support

There are several psychological and behavioral economic theories that exist in the literature; however, the pain of paying provides a solid theoretical foundation for the concept of Spendception, which is based on it. Both the conceptualization of Spendception and the construction of its measurement dimensions are supported by these theories, which provide useful insights into the ways in which the shape and visibility of money influence consumer behavior.

### Pain of Paying

Ariely and Prelec (Ariely & Prelec, 2003) presented the idea of the “pain of paying”, which is a term that defines the psychological discomfort that is linked with spending money. According to this hypothesis, different means of payment elicit different levels of cognitive and emotional participation during transactions, and these levels vary from person to person. Physical cash transactions include a concrete trade, in which persons physically part with money. Digital payments, on the other hand, require no money exchange at all. As a result of this visibility and tactility, the psychological pain of spending is amplified, and consumers become more conscious of the decisions they make regarding their finances. On the other hand, digital payment methods, such as credit cards or mobile wallets, abstract the process of spending, which lessens the emotional significance of the transaction and lessens the “pain”. Similarly, the word Spendception is a concept that encompasses the lessened psychological visibility and emotional detachment that are inherent with digital payments. This concept aligns well with the pain of paying idea.

## 4. Conceptual Framework

The contemporary era of digital payments provides fertile ground for impulse buying, a phenomenon characterized by sudden, impulsive, and unplanned purchasing decisions (Kong et al., 2025). Spendception is a construct that is fundamental to this behavior. It encompasses the convenience of digital payments, emotional detachment, perceived spending control, and diminished psychological visibility of spending. These dimensions collectively diminish the psychological barriers that have been traditionally associated with spending, thereby fostering an environment in which impetuous decisions are encouraged. The act of making a purchase becomes nearly frictionless when combined with the perceived ease and convenience of digital payments, which necessitate minimal effort or contemplation. This frictionless experience reduces the cognitive and emotional defenses that typically regulate consumer behavior, thereby inducing impulse purchasing.

**Figure 1:**
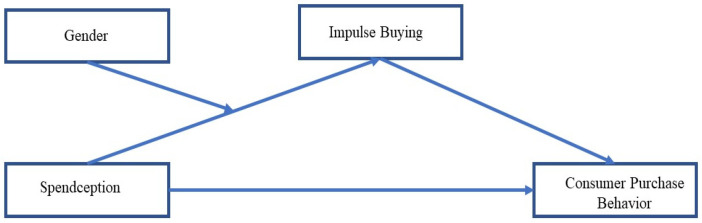
Conceptual model.

Consequently, impulse buying serves as a conduit between Spendception and an increase in purchasing behavior (Gupta et al., 2024; Kathuria & Bakshi, 2024; Melati et al., 2024). The psychological and emotional changes that Spendception introduces, such as reduced spending awareness and detachment, establish a favorable psychological environment for impulsive purchases. Consumers are not only more likely to purchase on impulse, but they are also more likely to justify these behaviors as insignificant or justified. This results in a cumulative increase in purchase behavior over time, which is influenced by the emotional detachment, invisibility, and convenience of digital payments. Figure 1 is showing the basic research model.

### 4.1. Rationales of the Study

This study on Spendception was motivated by the rapid global adoption of digital payment systems and their profound yet understudied psychological impact on consumer behavior. While traditional theories, like pain of paying, explain cash-based spending, they fail to address how the invisibility and frictionless nature of digital payments reduce psychological resistance to spending. This research fills this critical gap by introducing Spendception. Additionally, the study highlights gender differences. By combining advanced methodologies like SEM and machine learning, the research provides robust empirical evidence while offering practical implications for marketers, policymakers, and fintech firms to design ethical, consumer-centric payment systems. Ultimately, this work bridges theoretical gaps, addresses real-world behavioral challenges, and contributes actionable insights to promote mindful spending in the digital age.

### 4.2. Significance of the Study

This research contributes to the emerging field of consumer psychology in the digital payment context by introducing and empirically validating the concept of Spendception. It underscores the psychological and emotional mechanisms that drive modern spending behavior, offering valuable insights for policymakers, financial institutions, and businesses. By understanding these dynamics, stakeholders can devise strategies to mitigate overspending while leveraging the benefits of digital payments to foster sustainable consumer habits.

### 4.3. Novelty of the Study

This study introduces Spendception as a novel construct that captures the psychological and behavioral shifts in consumer spending driven by digital payment systems. Unlike prior research on the cashless effect and the pain of paying, which focus on general spending behavior, Spendception uniquely emphasizes the emotional detachment and perceived control that arise from the diminished visibility of digital transactions. It is the first to integrate these psychological mechanisms with impulse buying and gender differences, offering a comprehensive framework for understanding how digital payments reshape consumer behavior. By bridging gaps in consumer psychology and behavioral economics, Spendception provides a fresh perspective on the interplay between payment methods, emotional responses, and spending decisions. This pioneering contribution not only advances theoretical knowledge but also opens new avenues for research in the rapidly evolving digital payment landscape.

### 4.4. Hypotheses

**H1.**  *Spendception has a significant and positive relation with Impulse buying.*

**H2.**  *Impulse buying has a significant and positive relation with purchase behavior.*

**H3.**  *Spendception has a significant and positive relation with purchase behavior.*

**H4.**  *Impulse buying mediates between Spendception and purchase behavior.*

**H5.**  *Gender moderates the relationship between Spendception and purchase behavior mediated by impulse buying, such that females are more inclined to show impulsive buying when compared to males.*

## 5. Methodology

### 5.1. Survey Development

The survey was designed based on established theory in consumer psychology. Constructs such as Spendception were operationalized using a combination of adapted scales from prior research (Ariely & Prelec, 2003) and newly developed items to capture the unique psychological effects of digital payments. This study targeted 1162 Shanghai residents who completed a digital payment usage questionnaire. The dense population and advanced digital infrastructure of Shanghai, with over 24 million citizens, make it ideal for investigating digital payment consumer behavior. With official encouragement and widespread acceptance of Alipay and WeChat Pay, the city has led the way in digital payment systems. These technologies have changed residents’ payment patterns from cash to digital. 

Using Shanghai as the study location revealed how metropolitan policies, technology, and consumer preferences influence the uptake of digital payments. Ages, genders, marital situations, and income were covered in the survey, with ages from under 18 to 65 years.

### 5.2. Developing Questionnaire and Data Collection

We created an online questionnaire, given in Table 1, to examine how Spendception and impulse buying affect digital payment users’ shopping behavior. Spendception (psychological visibility of spending, perceived spending control, and simplicity of digital payments), impulse buying (Kong et al., 2025), and consumer purchase behavior were covered in the carefully crafted questions to meet the research objectives (Kim & Park, 2013). The questionnaire also asked about consumers’ emotional and psychological responses to digital payments and how these affect their purchases. A pilot test was conducted with 50 participants to assess the clarity and reliability of the survey. Based on their feedback, minor revisions were made to improve readability and reduce ambiguity.

The questionnaire was developed to eliminate ambiguity and misleading statements. To accurately assess respondents’ perspectives, a Likert scale from “strongly agree” (7) to “strongly disagree” (1) was used. This provided exact and comparative data collection on aspects of consumer behavior, such as digital payment psychological effects, impulse buying triggers, and spending patterns. 

Convenient sampling allowed the survey to reach a large audience quickly and cheaply. To acquire a complete and timely overview of consumer behavior trends, data were collected from January 2025. 

To improve collaboration and data quality, survey respondents were given extensive explanations of the questionnaire’s goal, importance, and technique. A comprehensive data filtering process removed incomplete or inconsistent responses to assure authenticity and validity. The study of 1162 valid questionnaires revealed a relationship between Spendception, impulse buying, and consumer purchase behavior. All participants supplied informed consent before participating in the study. The study followed the Declaration of Helsinki and was approved by the Ethics Committee of the University of Shanghai for Science and Technology. The research was approved by an IRB on 12 December 2024. All participants gave written informed consent. Participants gave informed consent through voluntary and confidential responses.

### 5.3. Data Analysis Methods

Data analysis was conducted in multiple stages to ensure robust and reliable results. First, exploratory factor analysis (EFA) was performed using principal component analysis with varimax rotation to identify the underlying structure of the constructs and validate the measurement model. Items with factor loadings below 0.5 were removed to ensure construct validity. Next, confirmatory factor analysis (CFA) was employed to confirm the factor structure, with model fit indices (CFI = 0.95, TLI = 0.94, and RMSEA = 0.06) indicating a good fit. Structural equation modeling (SEM) was then used to test the hypothesized relationships, including the mediating role of impulse buying and the moderating effect of gender. Finally, to validate the robustness of the model, machine learning techniques were applied, confirming the predictive power of the constructs. All analyses were conducted using SPSS-26 for EFA, AMOS-26 for SEM, and Python community edition 2024.1.1 for machine learning. Also, while preparing this article, we used Grammarly to enhance language and readability. Following this tool, we thoroughly reviewed and edited the content as necessary.

## 6. Results

Table 2 shows that the Cronbach’s alpha coefficients for all constructs were above 0.9, indicating a high level of internal consistency reliability. The Cronbach’s alpha coefficient of the overall scale was also high, at 0.953, indicating the reliability of the measurement results.

Table 3 shows the basic statistical characteristics of each variable. The minimum, maximum, mean, and standard deviations are given. In data preprocessing, descriptive statistical analysis was performed on the data using SPSS. This helped to understand the basic characteristics and distribution of the data.

Table 4 presents an overview of the participants’ basic information, including age, gender, and marital status. In terms of age distribution, the proportion of participants aged 25–34 was the highest, reaching 41.73%. In terms of gender, 56.2% were women. Married accounted for 58.27% of the marital status

The Kaiser–Meyer–Olkin (KMO) (Cerny & Kaiser, 1977; Shrestha, 2021) measure assessed sample readiness for factor analysis. Values closer to 1 indicate high variable correlations, indicating factor analysis suitability. This study’s KMO value was 0.939, which suggests that the data are suitable for factor analysis.

Table 5 presents the factor loading, reliability, and average variance extraction of each variable, which helped to evaluate the validity and reliability of the variable measurements.

Table 6 shows the correlations between the main components. The correlation between Spendception and impulse buying was 0.626, and with CPB was 0.559; however, the correlation between impulse buying and CPB was 0.54, indicating that there was a positive correlation among the components (Table 6).

Table 7 presents the common degree value of each variable, which is used to measure the degree to which the variance of a variable can be explained by common factors. The initial common degree of all three variables was 1, and the common degree of extraction was between 0.6481 and 0.798. This shows that these variables can be effectively explained by common factors to some extent.

Table 8 evaluates the goodness of fit of structural equation models. Several fit metrics, such as CFI, NFI, IFI, TLI, GFI, RMSEA, Chi-square, and $\chi^{2}/df$, were used. The values of CFI, IFI, and TLI were 0.95, 0.950, and 0.942, respectively, meeting the good fitting criteria of 0.9. NFI was 0.878, GFI was 0.858, and RMSEA was 0.061, which met the corresponding fitting criteria. Chi-square was 1.605, which also met the good fitting requirement of 3, indicating that the overall fitting effect of the model was good. This indicates that the research was reasonable and effective in setting the relationship between the variables related to consumer behavior. Based on this model, the internal relationship among Spendception, impulse buying, and consumer purchase behavior can be further analyzed and explained.

Table 9 presents the evaluation metrics for the model’s performance on both the training and test datasets. The mean squared error (MSE), mean absolute error (MAE), and R-squared (R^2^) values were provided to assess the accuracy and generalization ability of the model. As shown in Table 8, the model demonstrated excellent predictive power with R-squared (R^2^) values of 0.9792 for both the training and test sets, indicating that the model explained approximately 97.92% of the variance in both datasets. The mean squared error (MSE) for the training set (0.0092) was slightly lower than that for the test set (0.0123), which is typical and suggests the model’s good generalization to new data. Similarly, the mean absolute error (MAE) values for the training (0.0293) and test (0.0344) sets were close, supporting the conclusion that the model did not overfit and maintained accuracy across different data subsets. The low MSE and MAE values indicate that the model’s predictions were close to the actual values, with only a slight increase in the error when applied to unseen test data, suggesting good generalization.

**Figure 2:**
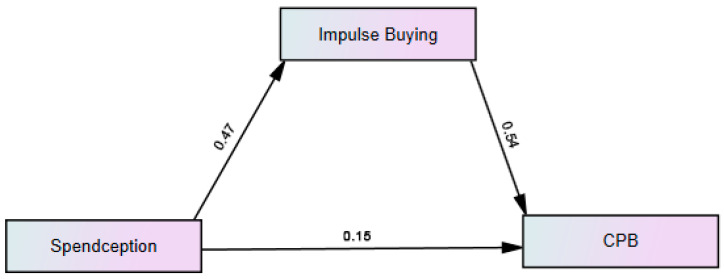
Direct and indirect paths.

Figure 2 shows a direct and indirect path relationship model between Spendception, impulse buying, and consumer purchase behavior.

Table 10 presents the direct effects of independent variables on the dependent variable:

**H1.**  *According to H1. The path coefficient (β-value) was 0.47 and the p-value was 0.005, and the result was accepted. This suggests that Spendception had a significant positive impact on impulse buying.*

**H2.**  *Impulse buying → consumer purchase behavior. The path coefficient was 0.544 and the p-value was 0.029, and the result was accepted. This suggests that impulse buying had a significant positive impact on consumer purchase behavior.*

**H3.**  *Spendception → consumer purchase behavior. The path coefficient was 0.15 and the p-value was 0.005, and the result was accepted. This indicates that Spendception had significant positive impact on consumer purchase behavior.*

Table 11 shows Spendception → IB → CPB (indirect effect), where the path coefficient was 0.252, *p*-value was <0.005, and the result was accepted. This suggests that impulse buying played a partial mediating role between Spendception and consumer purchase behavior.

The direct effect of the interaction term on IB was not significant. This implies that male gender had no direct moderating influence on the association between IB and Spendception. However, this finding warrants a deeper exploration into the psychological and social factors that may explain why male consumers did not exhibit a direct moderating influence. For instance, societal norms often encourage men to perceive themselves as rational decision-makers, which may suppress impulsive tendencies in certain contexts. This could explain why Spendception did not directly influence IB among male consumers.

### Indirect Effects of Spendception on CPB Through IB for Male Consumers

Table 12 examines how various degrees of male identity affected the indirect impacts of Spendception on CPB through IB.

Low male identity: The indirect impact was 0.087 with a SE of 0.086 at low levels of male identity, and it was significant at the *p* < 0.001 level (shown by ***). This indicates that when male identity was low, the relationship between Spendception, IB, and CPB was robust and substantial. This could be attributed to the fact that individuals with low male identity may feel less constrained by traditional gender roles, allowing Spendception to more easily trigger impulsive and consumer purchase behaviors.

Medium male identity: The indirect impact rose marginally to 0.158 with a SE of 0.051 at medium levels of male identity, and it was still significant, with a *p*-value of 0.003. This implies that, despite being weaker than at low male, the mediation pathway was nonetheless important, even at the medium male level. At this level, men may experience a tension between societal expectations of rationality and the emotional appeal of Spendception, leading to a moderate influence on IB and CPB.

High male identity: The indirect impact was 0.23 with a SE of 0.099 and a *p*-value of 0.099 at high levels of male identity, which was insignificant. This suggests that the mediation pathway weakened and moderation started playing its role; however, the index of moderate mediation had a *p*-value of 0.578, the SE was 0.035, and the moderated mediation index was −0.003, all of which were not significant. This implies that this model did not support the idea of moderated mediation. The lack of significance at high male identity levels may reflect the strong internalization of traditional masculine norms, which prioritize self-control and rational decision-making. This could act as a psychological barrier, reducing the impact of Spendception on IB and CPB among men with high male identity.

In contrast, the interaction between female identity and Spendception had a significant impact on impulse buying (IB), with an estimate of 0.034, SE of 0.014, and a critical ratio of 2.527, with a *p*-value of 0.012. This implies that there was a direct moderating influence when IB was decreased and both female consumer and Spendception were high. This finding highlighted the importance of considering gender-specific psychological and social factors in understanding consumer behavior.

Conditional Indirect Impacts: Table 13 examines how varying degrees of female identity affected the indirect impacts of Spendception on CPB through IB.

Low female identity: The indirect impact was 0.055 at low levels of female identity, with a SE of 0.023. It was significant at the *p* < 0.001 level. This indicates that when the female identity level was minimal, there was a strong and significant pathway from Spendception to IB and, ultimately, to CPB. This could be due to the fact that women with low female identity may feel less pressure to conform to traditional gender roles, allowing Spendception to more easily influence their buying behavior.

Medium female identity: With a *p*-value of 0.001, the indirect effect was still significant at medium levels of female identity, rising to 0.77 with a SE of 0.047. This implies that the mediation pathway was still important at medium female identity levels, more so than at low levels. At this level, women may experience a balance between societal expectations of emotional expressiveness and self-control, leading to a moderate influence of Spendception on IB and CPB.

High female identity: The *p*-value was 0.71, the indirect impact was 0.087, and the SE was 0.099 for high levels of female identity. This suggests that the mediation pathway became insignificant, and it suggested moderated mediation.

Index of moderate mediation: The *p*-value was 0.019, the SE was 0.035, and the moderated mediation index was −0.009. This index’s significance suggests that there was moderated mediation, which means that the degree of female identity influenced the association between Spendception and CPB through IB.

The presence of moderated mediation suggests that Spendception had a direct impact on CPB, but that this influence was also influenced by female identity, which in turn influenced IB.

## 7. Discussion

A multifaceted paradigm known as “Spendception” was developed to describe the behavioral and psychological changes in consumer spending brought about by digital payment systems. The psychological visibility of spending, perceived control over spending, and the emotional separation that arises when consumers utilize digital payment systems instead of cash are the main concerns of Spendception, as this study made clear. The results of this study have significant implications for comprehending how digital payment systems impact consumer behavior in Shanghai, as these elements collectively impact consumer spending.

The data provided excellent support for hypothesis H3, which proposed that spending perception has a significant and positive relationship with purchasing behavior. Our finding that digital payments reduce the psychological visibility of spending and affect the consumer purchase behavior aligned with studies on the cashless effect (Schomburgk et al., 2024). Our core concept also aligned with the study of Schomburgk (Schomburgk et al., 2024). However, unlike prior research, including that of Schomburgk, which focuses on general spending behavior, Spendception specifically addresses the emotional detachment and perceived control that arise from digital transactions, offering a more nuanced understanding of how payment methods influence consumer psychology. According to the study, digital payments—especially those made with credit cards, smartphone apps, and internet platforms—lessen the psychological visibility of spending. Because they physically transfer money and watch as their cash reserves decrease, customers who pay with cash experience an instant emotional connection to the transaction. Spendception reduces the mental friction related to spending because digital payments do not provide this instantaneous, tactile input. Because digital payments are so convenient, people tend to underestimate their total spending, which raises the possibility of impulsive purchases. This psychological effect is more pronounced in Shanghai’s highly digitalized economy, where online purchasing platforms, like Taobao, JD.com, and WeChat Pay, rule the market. Shanghai’s consumers, especially the younger ones, are used to the ease of quick transactions without the immediate repercussions. The impact of digital payment methods on consumer spending patterns is thus confirmed by the fact that “Spendception” leads to a notable rise in purchasing behavior.

H2 proposed that impulse shopping has a significant and positive relationship with purchasing behaviors, and the results of this study supported this hypothesis. The mediating role of impulse buying in our study was consistent with research by Coelho (Coelho et al., 2023), who found that frictionless payment systems increase unplanned purchases. However, Spendception goes further by explaining why this occurs, through the diminished visibility of spending and the emotional separation created by digital payment systems. This is a significant departure from prior studies, which often treat impulse buying as a standalone phenomenon without linking it to the payment method psychology. Digital payment systems, such as mobile apps and credit cards, exacerbate this effect by diminishing the psychological visibility of spending. Unlike cash transactions, where consumers are physically aware of the money leaving their wallets, digital payments create a psychological detachment from the act of spending, making consumers less aware of the financial consequences of their purchases. In Shanghai, where e-commerce and mobile payments are ubiquitous, the ease and convenience of digital transactions encourage consumers to act on impulse more frequently. Furthermore, high exposure to advertisements, promotions, and limited-time offers on shopping platforms like Taobao and JD.com often triggers these impulse-buying behaviors. The study also highlights that consumers, especially those in the 25–34 age group, are particularly susceptible to such influences, leading to purchase behavior. Overall, impulse buying plays a crucial role in driving unplanned purchases in the digital age, especially when consumers are exposed to Spendception tactics that reduce the psychological barriers to spending.

H4, which examined the function of impulse buying as a mediator between Spendception and purchase behavior, was also supported by the findings. As anticipated, the association between Spendception and consumer purchasing behavior was largely mediated by impulse buying. The simplicity of digital payments eases the cognitive burden of decision-making, enabling customers to make impulsive purchases. Spendception makes spending less visible, which encourages people to make impulsive purchases without thinking through the long-term financial effects. In Shanghai’s fast-paced consumption culture, where people are constantly exposed to online advertisements, promotions, and limited-time deals, this finding is consistent with the growing importance of impulse buying. Spendception in this context encourages impulsive buying, which contributes to higher consumer expenditure. The shift from Spendception cues to real purchase decisions is thus facilitated by impulse buying, which serves as a crucial mediator.

Spendception, consumer purchase behavior, and impulse buying are all affected by gender. According to H5, women are more likely than men to use digital payments and buy things online, which was also true in our study. Our result that women are more likely to buy things on impulse due to the concept of Spendception is similar to what Apriliana (Apriliana et al., 2024) found about how men and women spend money emotionally. However, our study is the first to link this vulnerability to the psychological workings of digital payments. This gives us a new way to look at how gender affects payment systems. What does it mean that men and women spend differently?

### 7.1. Implications of Gender Differences in Spending Behavior

The study’s results indicated that spending had a big effect on impulse buying (IB) and consumer purchase behavior (CPB), but the effects were different for men and women. It is not just a matter of numbers—these differences showed deeper psychological and social factors that affected how men and women react to marketing messages.

#### Psychological Factors

Emotional Regulation: Women are often taught to show their feelings more openly, which could make them more open to the emotional pleas in Spendception. This fits with previous studies (Pan et al., 2025; Rick et al., 2014; Xie et al., 2025) that showed that women are more likely to use retail therapy as a way to deal with stress or bad feelings.

Self-Control and Rationality: Men, on the other hand, are often told to be practical and self-controlled when making choices. This might be the reason why the direct effect of being a man on IB was not relevant. One study found (Hedhli et al., 2016) that men were more likely than women to see shopping as a task-oriented pastime rather than an emotional one.

Our study focused on Shanghai, where over 95% of payments are made digitally, and Spendception may differ in other cultures, economies, and policies. For example, the psychological effects of digital payments may be different in places like South Asia, where cash is still common because people are less familiar with and do not believe in digital systems. Spendception’s effects could be weakened even more by cultural factors like collectivism, fear of risk, and financial knowledge (Huang, 2023). Policy frameworks and economic factors, such as income levels and digital infrastructure, may also affect how digital payments are used and how they affect people’s minds. To get a better sense of this phenomenon, future studies should look at Spendception in various places, comparing highly digitalized economies (like China and Sweden) with cash-dominant regions (like South Asia and some parts of Africa).

## 8. Theoretical Contribution

This study makes several key theoretical contributions, as follows:

1. First, we introduced Spendception as a novel construct that bridges gaps in consumer psychology and behavioral economics by explaining how digital payment systems reduce psychological barriers to spending.
2. Second, we extended the pain of paying theory by demonstrating how the invisibility and emotional detachment of digital transactions reshape consumer behavior.
3. Third, we highlighted the mediating role of impulse buying and the moderating role of gender, offering new insights into the psychological mechanisms underlying digital payment-driven spending.

Furthermore, our finding that Spendception significantly increased purchase behavior aligns with and extends the cashless effect (Schomburgk et al., 2024).

Unlike previous studies that treat impulse buying as a standalone phenomenon (Coelho et al., 2023), our research demonstrated how it mediates the relationship between Spendception and purchase behavior, offering a more nuanced understanding of digital payment-driven spending.

## 9. Conclusions

This study set out to explore the intricate relationship between Spendception, impulse buying (IB), and consumer purchase behavior (CPB), with a particular focus on the moderating role of gender. Against the backdrop of rapidly evolving digital payment systems and their profound influence on consumer behavior, this research addressed a critical gap in the literature: the lack of understanding of how psychological and social factors, such as gender, shape the impact of modern payment methods on purchasing decisions. This study sought to answer the central research question: how does Spendception influence impulse and consumer purchase behaviors, and to what extent does gender moderate these relationships?

To answer this question, the study employed a robust methodological framework, combining structural equation modeling (SEM) and moderated mediation analysis to examine the direct and indirect effects of Spendception on IB and CPB, while accounting for gender differences. The findings revealed that Spendception significantly lowered the psychological barriers to spending, thereby enhancing impulse buying behavior, which in turn mediated the relationship between Spendception and consumer purchase behavior. These results validated the proposed theoretical framework.

One of the most impactful findings of this study was the significant moderating role of gender in the relationship between Spendception and impulse buying. Specifically, female consumers were found to be more susceptible to the effects of Spendception, likely due to societal norms that encourage emotional expressiveness and retail therapy among women. In contrast, male consumers exhibited a weaker response, reflecting the internalization of traditional masculine norms that prioritize self-control and rationality. The findings of this study have significant implications for both businesses and policymakers. For businesses, the results underscore the importance of designing gender-sensitive marketing strategies that account for the differing psychological and social influences on male and female consumers.

For policymakers, the study highlighted the need for consumer protection measures to safeguard vulnerable populations, particularly women, from exploitative marketing practices.

In conclusion, this study shed light on the complex interplay between digital payment methods, psychological factors, and consumer behavior. By validating the proposed theoretical framework and uncovering the moderating role of gender, we provided a nuanced understanding of how modern payment systems influence purchasing decisions. The findings not only advance academic knowledge but also offer actionable insights for businesses and policymakers, making this research relevant to a broad audience, including industry professionals and consumer advocates. As digital payment systems continue to evolve, understanding their psychological and social implications will remain a critical area of inquiry for researchers and practitioners alike.

## 10. Practical Implications

The findings of this study offer significant practical implications for businesses, policymakers, and consumers. For marketers, understanding Spendception provides a strategic advantage in designing digital payment interfaces and promotional campaigns that leverage reduced psychological friction to drive sales. For instance, platforms like Taobao and WeChat Pay can use personalized recommendations and limited-time offers to capitalize on impulse buying tendencies. For policymakers, these insights highlight the need for regulations that promote financial literacy and transparency in digital transactions, such as real-time spending alerts or budgeting tools, to mitigate overconsumption. For consumers, awareness of Spendception can encourage more mindful spending habits, particularly among vulnerable groups like younger demographics and women, who are more susceptible to digital payment-driven impulsivity. By addressing both the opportunities and risks associated with Spendception, this study provides actionable insights for fostering a balanced and ethical digital payment ecosystem.

### 10.1. Advice for Marketers

Use of Spendception in marketing: Marketers can employ Spendception to boost impulse purchases. The rapid, flawless, and psychologically detached purchasing procedure lowers the perceived cost of spending. One-click payments, mobile payment systems, and other payment methods should be prioritized by digital platforms to help consumers make faster purchases. They should guarantee these approaches are ethical to minimize consumer displeasure and reaction.

Target female emotional triggers: Since female consumers are more sensitive to Spendception and impulse buying, marketers should use emotional appeals and targeted offers. These may include limited-time deals, targeted discounts, or social proof (e.g., influencer endorsements) to attract female shoppers, who are more emotional about purchases. H5 showed that males are less sensitive to Spendception, thus marketers should promote financial transparency and spending control options to men. For cautious and budget-conscious men, campaigns could include financial management applications, spending alerts, or savings awards.

Provide digital payment system expense tracking: Digital payment platforms should include expense tracking to assist users’ budgets. Marketers can help consumers avoid overspending by presenting monthly expense summaries or spending categories. The moderating effect of Spendception may encourage more appropriate consumer behavior by making spending visible.

### 10.2. Advice for Consumers

Beware of psychological spending traps: Customers should be informed of the psychological repercussions of digital payments, such as reduced payment pain and financial isolation. Understanding Spendception helps consumers set spending limits, use budgeting tools, and track financial objectives to avoid impulsive purchase. Digital solutions, like expense tracking applications, budgeting tools, and spending limit alerts, can help consumers cut back on spending. These tools can give them a more precise financial picture, enabling them to make informed decisions without Spendception.

Mindful spending: Consumers should pause before buying, especially with online discounts. They should set a monthly budget, manage expenditures, and limit impulse spending to reduce Spendception and help consumers reach their financial goals.

## 11. Future Research Directions

It is crucial to acknowledge the counterbalancing effect of the continuous tracking of expenditures made possible by digital payment systems, even though Spendception encourages consumer purchase behavior by lowering the psychological barriers related to spending. Digital payments give customers monthly expenditure records, which are frequently shown through mobile applications or online banking platforms, in contrast to cash payments, which are instantaneous and do not directly record previous transactions. As customers grow more conscious of their accrued expenses, this visibility of spending may operate as a moderating factor, possibly restricting buying behavior. As customers examine their monthly expenses, the ability to monitor spending trends over time may result in increased financial consciousness, which may drive them to reevaluate their purchases. Therefore, the recording and visibility of these payments may serve as a moderating influence, encouraging more cautious spending behavior and thereby reducing impulsive purchases, even though Spendception may initially lessen the perceived cost of individual transactions. As a crucial element in comprehending consumer spending patterns, our future research will concentrate on the creation and investigation of cost tracking systems. We intend to examine how ongoing monitoring and visibility of spending can affect customers’ decision-making processes, especially in reducing impulsive purchases, by utilizing the data offered by digital payment networks. In order to develop more effective strategies for controlling consumer spending behavior in the digital age, this field of study will help us better understand the balance between the psychological ease of digital payments, which is driven by Spendception, and the moderating effects of expense awareness.

## Figures and Tables

**Table 1:** Statistics of the measurement items for consumer inner states.

Variable	Coding	Measurement Items
Spendception	SP1	When I pay with cash, I feel more aware of how much I am spending, as compared to digital payment
	SP2	Digital payments make it harder for me to realize how much money I have spent
	SP3	Seeing physical cash leave my hand makes me think twice before spending
	SP4	Paying with digital methods feels less painful than paying with cash
	SP5	I feel like I spend more when I do not see the money physically leaving my wallet
	SP6	Digital payments make spending feel easier and less stressful
	SP7	I do not feel the pain of spending when I pay digitally compared to cash
	SP8	When I pay with digital methods, it feels like I am not really spending money
	SP9	I feel more in control of my spending when I pay with cash
	SP10	I feel like I lose control over my budget when I rely on digital payments
	SP11	Paying with cash helps me stick to my spending limits better than digital payments
	SP12	The convenience of digital payments makes me more likely to spend
Impulse Buying	IB1	Digital currency makes it easier to buy items I had not planned to purchase
	IB2	I am more likely to make impulsive purchases when using digital payments
	IB3	The convenience of digital currency encourages unplanned spending
	IB4	I tend to spend more on impulsive purchases with digital currency than with cash
	IB5	Digital payments reduce my hesitation to buy items on impulse
Consumer Purchase Behavior	CPB1	I purchase more frequently now because digital currency makes transactions easier
	CPB2	I spend more on monthly purchases since adopting digital currency
	CPB3	I am more likely to purchase non-essential or luxury items because digital currency is convenient
	CPB4	Digital currency has made me more confident in spending larger amounts
	CPB5	My shopping habits have changed significantly due to digital currency

**Table 2:** Cronbach’s alpha table.

Construct	Number of Items	Cronbach’s Alpha	High Reliability Interpretation
Spendception	12	0.972	High Reliability
Impulse Buying	5	0.984	High Reliability
Consumer Purchase Behavior	5	0.903	High Reliability
Total	22	0.953	High Reliability

**Table 3:** Descriptive statistics.

	Minimum	Maximum	Mean	Standard Deviation
Spendception	1	7	6.3704	0.70074
Impulse Buying	1	7	5.2457	0.666
Consumer Purchase Behavior	1	7	5.3292	0.60306

**Table 4:** Participants’ profiles (N = 1162).

Item	Description	Frequency	Percentage
Age	Under 18	4	0.3
	18–24	239	20.5
	25–34	485	41.73
	35–44	309	26.6
	45–54	85	7.3
	Above 54	40	3.57
Gender	Male	509	43.8
	Female	653	56.2
Marital Status	Married	677	58.27
	Unmarried	485	41.73

**Table 5:** Factor loadings and reliability analysis, average variance extraction, and composite reliability.

Variables	Items	Loading	Cronbach’s α	CR	AVE
Spendception	SP1	0.581	0.972	0.876	0.589
	SP2	0.766			
	SP3	0.841			
	SP4	0.784			
	SP5	0.837			
	SP6	0.876			
	SP7	0.747			
	SP8	0.720			
	SP9	0.683			
	SP10	0.909			
	SP11	0.788			
	SP12	0.459			
Impulse Buying	IB1	0.742	0.972	0.880	0.556
	IB2	0.882			
	IB3	0.808			
	IB4	0.809			
	IB5	0.589			
Consumer Purchase Behavior	CPB1	0.886	0.903	0.915	0.783
	CPB2	0.867			
	CPB3	0.901			
	CPB4	0.891			
	CPB5	0.786			
Total			0.953		

**Table 6:** Component correlation matrix.

Components	Spendception	Impulse Buying	CPB
Spendception	1		
Impulse Buying	0.626	1	
CPB	0.559	0.540	1

**Table 7:** Commonality values.

Variables	Initials	Extraction
Spendception	1	0.679
Impulse Buying	1	0.6481
Consumer Purchase Behavior	1	0.664

**Table 8:** Goodness of fit.

Fit Indices	Definition	Criteria	Values
CFI	Comparative fit index	>0.9 good fit	0.95
NFI	Normed fit index	>0.9 good fit	0.878
IFI	Incremental fit index	>0.9 good fit	0.950
TLI	Tucker–Lewis index	>0.9 good fit	0.942
GFI	Goodness of fit	>0.9 good fit	0.858
RMSEA	Root mean squared error of approximation	0.08 > good fit	0.061
$\chi^{2}/df$	Chi-square	3 > good fit	1.605

**Table 9:** Machine learning model performance evaluation: training and test set metrics.

Metric	Train Set	Test Set
Mean squared error (MSE)	0.0092	0.0123
Mean absolute error (MAE)	0.0293	0.0344
$R-\mathrm{squared}\;\left(R^{2}\right)$	0.9792	0.9792

**Table 10:** Hypothesis results.

Hypotheses	Path Direction	β-Value	*p*-Value	Result
H1	Spendception → Impulse Buying	0.47	***	Accepted
H2	Impulse Buying → Consumer Purchase Behavior	0.54	0.029	Accepted
H3	Spendception → Consumer Purchase Behavior	0.15	***	Accepted

*** Significant at 0.001 < *p* < 0.005.

**Table 11:** Mediation analysis.

Hypotheses	Path Direction	β-Value	*p*-Value	Result
	Spendception → CPB	c=0.47	***	
	Spendception → CPB (Direct Effect)	$c^{/}=0.152$	0.073	
	IB → CPB	0.539	***	
H4	Spendception → IB → CPB	0.252	***	Accepted

*** Significant at 0.001 < *p* < 0.005.

**Table 12:** Direct and indirect moderating effects of male consumers through the Hayes process.

Path	Estimate	SE	Critical Ratio	*p*-Value	Interpretation
Direct Effects					
Spendception → IB	0.382	0.101	3.773	0.020	Spendception positively and significantly influences male identity
Male → IB	0.149	0.147	3.049	0.002	Male identity moderately enhances IB
Interaction_Male_Spendception → BC	−0.1	0.04	0.713	0.476	Interaction effect is weak and insignificant, indicating no direct moderation
IB → CPB	0.327	0.043	7.741	***	PBC drives BEIV
Spendception → CPB	0.359	0.41	8.670	0.029	Spendception also has a significant direct impact on CPB
Conditional Indirect Effects					
Low Male Identity	0.087	0.086	N/A	***	The indirect pathway (Spendception → IB → CPB) is strong and significant at low male identity
Medium Male Identity	0.158	0.051	N/A	0.003	The mediation pathway weakens but remains significant at the medium male identity level
High Male Identity	0.23	0.099	N/A	0.099	At the high male identity level, the mediation pathway becomes insignificant
Moderated Mediation Index	−0.003	0.035	N/A	0.578	The insignificant index indicates that moderated mediation does not exist

*** Significant at 0.001 < *p* < 0.005.

**Table 13:** Direct and indirect moderating effects of female identity through the Hayes process.

Path	Estimate	SE	Critical Ratio	*p*-Value	Interpretation
Direct Effects					
Spendception → IB	0.521	0.094	5.546	***	Spendception significantly and positively influences IB
Female → IB	0.732	0.150	4.892	***	Female identity significantly and positively influences IB
Interaction_Female_Spendception → IB	0.034	0.014	2.527	0.012	Interaction term significantly influences IB, indicating direct moderation
IB → CPB	0.275	0.040	6.666	***	IB significantly and positively influences CPB
Spendception → CPB	0.451	0.036	12.406	***	Spendception significantly and positively influences CPB
Conditional Indirect Effects					
Low Female Identity	0.055	0.023	N/A	***	The indirect pathway (Spendception → IB → CPB) is strong and significant at low female identity
Medium Female Identity	0.77	0.047	N/A	0.001	The mediation pathway weakens but remains significant at medium female identity
High Female Identity	0.087	0.099	N/A	0.07	At high female identity levels, the mediation pathway weakens and is insignificant, which suggests moderation
Moderated Mediation Index	−0.009	0.035	N/A	0.019	The significant index indicates that moderated mediation exists

*** Significant at 0.001 < *p* < 0.005.

## Data Availability

The data presented in this study are contained within the article and available on request.
